# Early Growth Response 4 Is Involved in Cell Proliferation of Small Cell Lung Cancer through Transcriptional Activation of Its Downstream Genes

**DOI:** 10.1371/journal.pone.0113606

**Published:** 2014-11-20

**Authors:** Taisuke Matsuo, Le Tan Dat, Masato Komatsu, Tetsuro Yoshimaru, Kei Daizumoto, Saburo Sone, Yasuhiko Nishioka, Toyomasa Katagiri

**Affiliations:** 1 Division of Genome Medicine, Institute for Genome Research, The University of Tokushima, Tokushima, Japan; 2 Department of Medical Oncology, Institute of Health Biosciences, The University of Tokushima, Tokushima, Japan; Univesity of Texas Southwestern Medical Center at Dallas, United States of America

## Abstract

Small cell lung cancer (SCLC) is aggressive, with rapid growth and frequent bone metastasis; however, its detailed molecular mechanism remains poorly understood. Here, we report the critical role of early growth factor 4 (EGR4), a DNA-binding, zinc-finger transcription factor, in cell proliferation of SCLC. EGR4 overexpression in HEK293T cells conferred significant upregulation of specific splice variants of the parathyroid hormone-related protein (*PTHrP*) gene, resulting in enhancement of the secretion of PTHrP protein, a known mediator of osteolytic bone metastasis. More importantly, depletion of *EGR4* expression by siRNA significantly suppressed growth of the SCLC cell lines, SBC-5, SBC-3 and NCI-H1048. On the other hand, introduction of *EGR4* into NIH3T3 cells significantly enhanced cell growth. We identified four *EGR4* target genes, *SAMD5*, *RAB15*, *SYNPO* and *DLX5*, which were the most significantly downregulated genes upon depletion of *EGR4* expression in all of the SCLC cells examined, and demonstrated the direct recruitment of EGR4 to their promoters by ChIP and luciferase reporter analysis. Notably, knockdown of the expression of these genes by siRNA remarkably suppressed the growth of all the SCLC cells. Taken together, our findings suggest that EGR4 likely regulates the bone metastasis and proliferation of SCLC cells via transcriptional regulation of several target genes, and may therefore be a promising target for the development of anticancer drugs for SCLC patients.

## Introduction

Lung cancer is one of the most common cancers, and its incidence is rising worldwide [1]. The high mortality and poor prognosis of lung cancer result from difficulties in early diagnosis and its high metastatic potential. Lung cancer is classified into two major types, small-cell lung cancer (SCLC) and non-small cell lung cancer (NSCLC), which account for approximately 25% and 75% of cases, respectively. SCLC presents with aggressive clinical behavior characterized by rapid growth and frequent metastases to the brain, lung, liver and bone [2]. In particular, bone metastasis causes severe complications in SCLC and can lead to bone pain, pathological fractures, hypercalcemia, spinal cord compression and other nerve compression syndromes [3], [4], and it is often associated with high morbidity and poor prognosis. Current treatments are generally palliative. Therefore, it is highly important to prevent and treat osteolytic bone metastases.

Bone metastasis has been generally classified as osteolytic, leading to bone destruction; osteoblastic, leading to new bone formation; or mixed based on the primary mechanism of interference with normal bone remodeling. The balanced activity of osteolytic and osteoblastic factors is thought to regulate bone metastasis [4], [5]. Recently, several molecules have been reported to play important roles as osteoblastic factors involved in osteoformation [4]–[6]. However, the precise mechanisms responsible for tumor growth in bones remain unexplored.

Comprehensive transcriptomics confer a precise characterization of individual cancers that should help to improve clinical strategies for neoplastic diseases through the development of novel drugs. Hence, “omics” technology approaches are effective for identifying target molecules involved in carcinogenic and metastatic pathways, including bone metastasis. To this end, the genome-wide transcriptomics of human SCLC engaged in organ-preferential metastasis in mice was analyzed, and several genes potentially involved in bone metastasis were found [7]. In this study, we focused on early growth response 4 (*EGR4*), which is significantly upregulated in bone metastatic tumors compared with other organ metastases (lung, kidney and liver) derived from human SCLC cells [7].

The *EGR4* gene belongs to the early growth response family of immediate early genes encoding four DNA-binding, zinc-finger transcription factors (*EGR1* to *EGR4*) [8]. This gene (*pAT133*, *NGFI-C*) was first identified as a zinc-finger protein immediately induced by mitogenic stimulation in T lymphocytes and fibroblasts [9], [10]. It has been reported that *EGR4*-null mice have male infertility because of arrested spermatogenesis but no female infertility is observed [11], [12], suggesting that EGR4 plays a critical role in some types of human idiopathic male infertility. Moreover, EGR4 is known to have a neural-specific expression pattern in rats [13] and regulate brain-derived neurotrophic factor (BDNF)-mediated neuron-specific potassium chloride cotransporter 2 (KCC2) transcription via the ERK1/2 signaling pathway in immature neurons [14]. However, the pathophysiological role of EGR4 in carcinogenesis in SCLC, has not been elucidated. In this study, we report that EGR4 acts as a transcriptional activator via regulation of specific downstream genes in SCLC cell proliferation.

## Materials and Methods

### Cell lines

The human SCLC cell lines SBC-3 and SBC-5 were kindly provided by Drs. M. Tanimoto and K. Kiura of Okayama University [15]. The NSCLC cell line PC14PE6 was kindly provided by Dr. I. J. Fidler of M. D. Anderson Cancer Center [16]. The human SCLC cell line NCI-H1048 and human NSCLC cell lines A549 and NCI-H1048 were purchased from the American Type Culture Collection (ATCC, Rockville, MD, USA). The human ACC-LC319/bone2 cell line was established as previously described [17]. The MC3T3-E1 murine osteoblastic subclone 4 cell line was kindly provided by Chugai Pharmaceutical Co., Ltd. (Tokyo, Japan). The human small airway epithelial cell line (SAEC) was purchased from Lonza (Walkersville, MD, USA). All cells were cultured under appropriate conditions.

### Plasmid constructs

The entire coding sequence of human *EGR4* (NM_001965) was amplified by PCR using KOD plus DNA polymerase (Toyobo, Osaka, Japan). The PCR product was inserted into the *Eco*RI and *Xho*I sites of the pCAGGSn3FH vector which contains an N-terminal FLAG tag. For luciferase reporter plasmids, DNA fragments from the 5′-flanking regions of *PTHrP-V3* and *V4* (NM_198964.1 and NM_198966.1, respectively), *SAMD5* (NM_001030060.2), *RAB15* (NM_198686.2), *SYNPO* (NM_007286.5) and *DLX5* (NM_005221.5), which include potential EGR binding sites as predicted by the MatInspector program (Genomatix, http://www.genomatix.de/matinspector.html), were amplified by PCR and inserted into the appropriate restriction enzyme sites in the pGL3-enhancer vector (Promega, Madison, WI, USA). The PCR primer sets used in this study are shown in Table S1. The DNA sequences of all constructs were confirmed by DNA sequencing (ABI 3500xL sequencer; Life Technologies, Foster City, CA, USA).

### RNA extraction, reverse transcription, semi-quantitative PCR and real-time PCR

Total RNA extraction, reverse-transcription, semi-quantitative RT-PCR and Real-time PCR experiments were conducted as previously described [18]. The expression levels in each sample were normalized to the *β*-actin mRNA content. The sequences of each primer set are listed in Table S2.

### Western blot analysis

Western blot analysis was performed as previously described [18]. After SDS-PAGE, membranes blotted with proteins were incubated with anti-FLAG M2 (Sigma-Aldrich, St. Louis, MO, USA, F3165) or anti-β-actin (AC-15, Sigma-Aldrich, A-5441) mouse monoclonal antibodies diluted at 1∶5000. The membranes were then incubated with a horseradish peroxidase (HRP)-conjugated secondary antibody for 1 h, and the protein bands were visualized with enhanced chemiluminescence (ECL) detection reagents (GE Healthcare, Piscataway, NJ, USA).

### Measurement of PTHrP secretion

HEK293T cells (1.5×10^5^ cells/12-well plate) were transfected with the pCAGGSn3FH-EGR4 or mock (no insert) plasmids using FuGENE 6 (Promega). At 48 h after transfection, the culture medium was collected and centrifuged at 4°C at 15,000 rpm. The PTHrP protein concentration in the conditioned media was determined by an immunoradiometric (IRMA) assay (SRL Inc., Tokyo, Japan).

### Effect of conditioned medium derived from EGR4-overexpressing HEK293T cells on *RANLK*, *IL-6* and *IL-8* expression

HEK293T cells (2.6×10^6^ cells/10 cm plate) were transiently transfected with the pCAGGSn3FH-EGR4 or mock vector for 48 h, and the culture media was then replaced with DMEM plus 0.1% FBS for an additional 48 h. The culture medium was subsequently collected, and the conditioned medium was transferred to murine MC3T3-E1 osteoblast cells that were pre-cultured with differentiation medium containing ascorbic acid (100 µg/ml) for 5 days. After 48 h, the expression levels of murine *RANKL*, *IL-6*, and *IL-8* was analyzed by real-time PCR as described above.

### Chromatin immunoprecipitation (ChIP) assay

HEK293T cells (2.5×10^6^ cells/10 cm dish) were transfected with 8 µg of the pCAGGSn3FH-EGR4 or mock vector for 48 h and then ChIP assays were performed using the EZ-ChIP kit (Millipore, Billerica, MA, USA) as previously described [19]. The PCR primer sets to detect the EGR-binding sites used are listed in Table S3.

### Luciferase assay

HEK293T cells (2.5×10^4^ cells/48-well dish) were co-transfected with either 100 ng of the pGL3-enhancer promoter vector as described above or the mock vector in combination with 100 ng of the pCAGGSn3FH-EGR4 or mock vector (100 ng). pRL-TK was used as an internal control. After 48 h, the cells were harvested and analyzed for *Firefly* luciferase and *Renilla* luciferase activity using the dual luciferase reporter assay (Promega) as previously described [19]. Data were expressed as the fold increase over mock-transfected cells (set at 1.0) and represented as the mean ± SE of two independent experiments.

### NIH3T3 cell proliferation assay

NIH3T3 cells (0.5×10^5^ cells/6-well dish) were transiently transfected with 3 µg of pCAGGSn3FH-EGR4 or mock vector using FuGENE 6 (Promega). Cell proliferation assays were performed at 48, 72 and 96 h after transfection, respectively, using Cell Counting Kit-8 (Dojindo, Kumamoto, Japan) as previously described [18]. These experiments were performed in triplicate. Western blot analysis was performed as described above.

### Gene silencing effects by siRNA treatment

We used siRNA oligonucleotides (Sigma-Aldrich Japan KK, Japan) to knock down *EGR4*, *DLX5, SYNPO SAMD5* and *RAB15* expression in SBC-5, SBC-3, NCI-H1048 or PC14PE6. The sequences targeting each gene are listed in Table S4. Cells were plated in 12-well dishes (SBC-5 and PC14PE6; 1.5×10^4^ cells/well, SBC-3; 2.5×10^4^ cells/well, NCI-H1048; 5.0×10^4^ cells/well). Transfection of 100 nM siRNA to SBC-5 and PC14PE6 cells was performed using Lipofectamine 2000 reagent (Life Technologies) as previously described [20]. SBC-3 and NCI-H1048 cells were transfected with 50 nM siRNAs using Lipofectamine RNAi Max transfection reagent (Life Technologies) according to the manufacturer's instructions. At 48, 96 or 120 h after transfection, total RNA extraction, real-time PCR and cell proliferation assays were performed as described above.

### Identification of EGR4 downstream genes by DNA microarray

SBC-5 cells (1×10^6^ cells/35 mm dish for 24 h) were transfected with 10 nM siRNA directed against EGR4 (EGR4-2) or EGFP (siEGFP; a control) using Lipofectamine RNAi Max transfection reagent (Life Technologies). Total RNA was extracted from each sample at 48 and 72 h after transfection of siRNA. The DNA microarray and data analyses were performed using the Agilent Whole Human Genome Microarray (4×44K, G4110F; Agilent Technologies, Santa Clara, CA, USA) and GeneSpring software (version 11.5; Agilent Technologies) as previously described [21]. A corrected *P* value was calculated with Benjamini Hochberg false discovery rate (FDR) analysis, and *P*<0.05 was considered significant. The extent and direction of the differential expression between time points (48 and 72 h) were determined by calculating fold change values. The DNA microarray analysis data have been submitted to the NCBI Gene Expression Omnibus (GEO) database as series GSE40558.

### RNAseq data analysis of lung cancers

Publicly available gene expression data (normalized values from Illumina RNAseq v2, level 3, LUAD and LUSC) from The Cancer Genome Atlas (TCGA; http://cancergenome.nih.gov/) were downloaded from TCGA matrix. The differential expression (by fold change value) between cancer tissues and the adjacent normal lung was calculated according to the normalized gene expression value of each sample.

### Statistical analysis

Statistical analysis was performed using Student's *t*-test. *P*<0.05 was considered significant.

## Results

### EGR4 directly regulates the transcriptional activity of the *PTHrP* gene

Analysis of the genome-wide gene expression profile of the organ-preferential metastasis of the human SCLC cell line SBC-5 in mice identified early growth response 4 (*EGR4*), which was significantly upregulated in bone metastatic tumors (*p*<0.001, ratio; 2.22) compared with other organ metastases (lung, kidney and liver) [7]. First, to clarify the role of EGR4 as a transcription factor involved in bone metastasis, we focused on the parathyroid hormone-related protein (*PTHrP*) gene as a candidate downstream target of EGR4 because the *PTHrP* gene is known to be a potent activator of osteoclastic bone resorption [4] and encodes a protein secreted from SBC-5 cells [22], [23]. Moreover, it has been reported that treatment with an anti-PTHrP neutralizing antibody inhibits the production of SBC5 cell bone metastasis in the SCID mouse model [22], [23].

**Figure 1:**
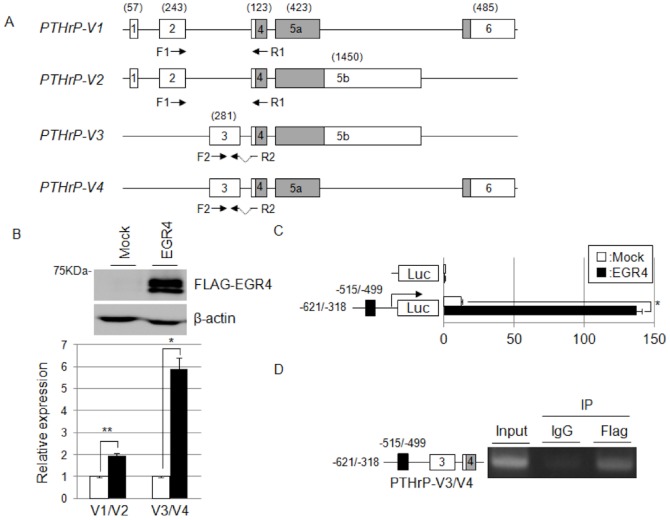
EGR4 directly transactivates specific splice variants of the *PTHrP* gene. A: Genomic structures of the four splice variants of *PTHrP*. The gray and white boxes indicate coding and non-coding regions, respectively. The arrows indicate the primer sets used to perform RT-PCR for each transcript. The numbers in parentheses indicate the length of each exon. B: Upper panel: western blot analysis of HEK293T cells expressing exogenous FLAG-tagged EGR4 (FLAG-EGR4) or cells transfected with the mock vector. Lower panel: real-time RT-PCR analysis of *PTHrP* splice variants (*V1/V2* and *V3/V4*) in EGR4-overexpressing HEK293T cells. C: Luciferase assay of the *PTHrP-V3* and *-V4* (*V3/V4*) promoter regions (n = 2, *P<0.05). This experiment was performed using a part of the lysates from cells expressing exogenous FLAG-EGR4 or those transfected with the mock vector used in B. D: ChIP assay of the *PTHrP-V3/V4* promoter region. ChIP assays were used to determine direct EGR4 binding to the *PTHrP-V3/V4* promoter. The PCR product was from −620 to −318 of the region upstream of the 5′ end of exon 3 of *PTHrP-V3/V4*, which was designated as the +1 position.

In the National Center for Biotechnology Information (NCBI) database, the *PTHrP* gene is reported to possess four transcriptional variants, designated *PTHrP* variant 1 (PTHrP-V1, GenBank accession no. NM_198965.1), variant 2 (PTHrP-V2, NM_002820.2), variant 3 (PTHrP-V3, NM_198964.1) and variant 4 (PTHrP-V4, NM_198966.1). The full-length cDNAs of *PTHrP-V1*, *V2*, *V3* and *V4* consist of 1331, 1881, 1862 and 1312 nucleotides that encode 177, 175, 175 and 177 amino acids, respectively, and consist of 5, 4, 3, and 4 exons, respectively. The V1 variant lacks exon 3, and the V2 variant lacks exon 3 and possesses exon 5b, which is 1,027 bp longer at the 3′ end than exon 5a. The V3 and V4 variants commonly lack exons 1 and 2 and possess exon 3, which is located within intron 2 with a length of 281 bp. The V3 variant further lacks exon 6, and possesses exon 5b and V2 variant. The V4 variant possesses exon 5a and exon 6, indicating that the *PTHrP-V1/V2* and *V3/V4* variants have different promoter regions (Figure 1A). Subsequent real-time RT-PCR analysis confirmed that the *PTHrP-V3* and *V4* splicing variants were predominately upregulated at the transcriptional level in EGR4-overexpressing HEK293T cells compared with mock-transfected cells (Figure 1B). Accordingly, to obtain direct evidence for the upregulation of *PTHrP-V3* and *V4* by EGR4, we first searched for putative EGR DNA binding motifs with the MatInspector program (described above) because it has been reported that the EGR family, including the EGR4 protein, preferentially binds to an EGR consensus motif (5′-GCGG/TGGGCG-3′) [24]–[27]. We found a potential EGR DNA binding motif within the *PTHrP-V3* and *V4* promoter region (−515 to −499). Subsequently, we examined the transcriptional activity of EGR4 by a luciferase reporter assay using a pGL3 luciferase plasmid containing the EGR4 binding motif in the *PTHrP-V3/V4* promoter. A significant increase in luciferase activity was observed with FLAG-EGR4 transfection compared with the mock control vector in HEK293T cells (Figure 1C). To further investigate whether EGR4 could bind to a potential *PTHrP-V3* and *V4* EGR binding motif, we performed a ChIP assay. The genomic fragment including the potential EGR binding motif (−515 to −499) of *PTHrP-V3* and *V4* was specifically bound by EGR4 protein in products immunoprecipitated with an anti-FLAG antibody, suggesting that EGR4 directly bound to the promoter region of the *PTHrP-V3* and *V4* variants (Figure 1D). Taken together, these findings suggest that the EGR4 might directly upregulate the *PTHrP-V3* and *V4* variants in SCLC cells.

### Paracrine effects of PTHrP secreted from EGR4-overexpressing cells

**Figure 2:**
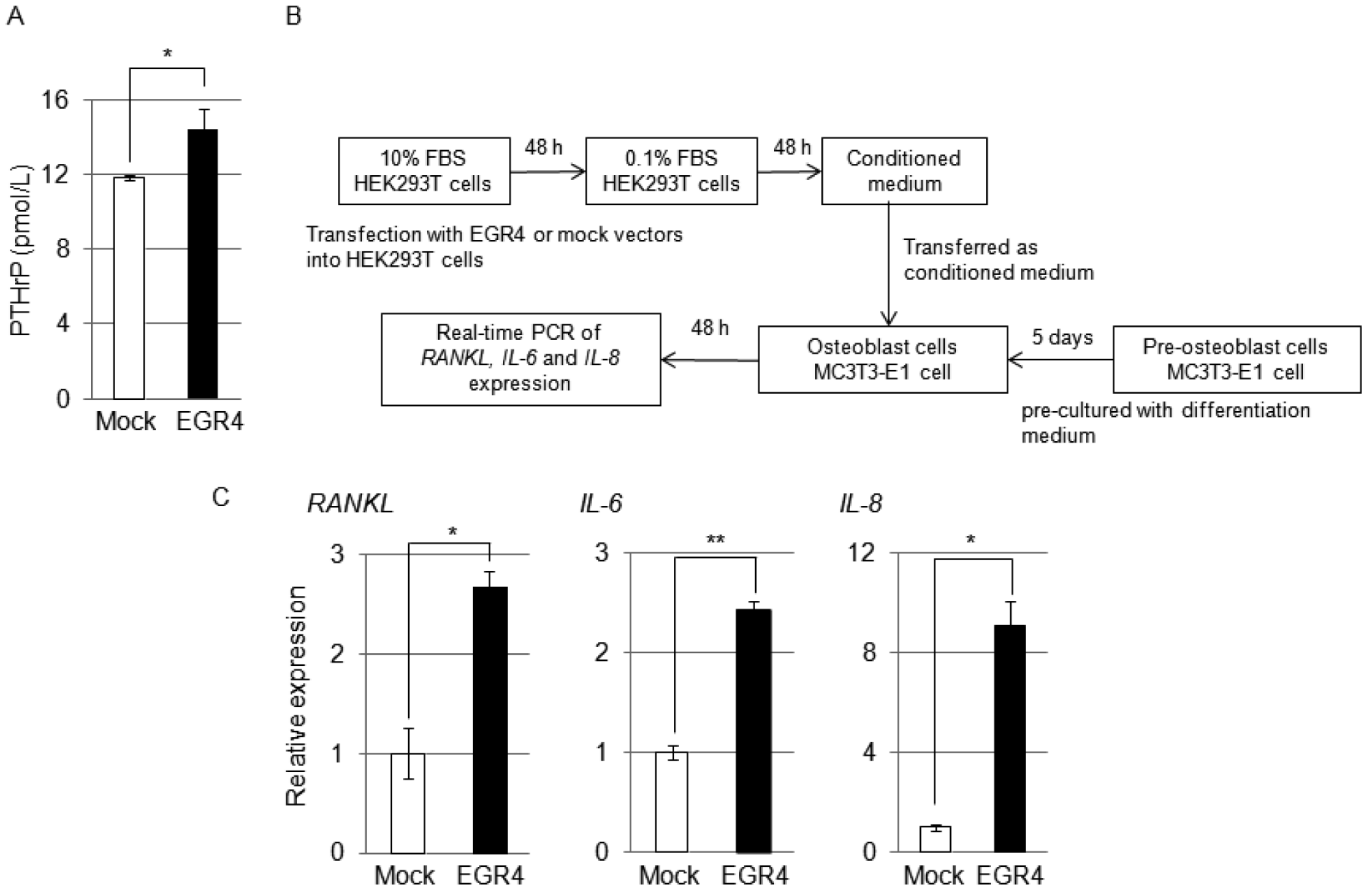
PTHrP secretion leads to transactivation of specific *PTHrP* splice variants. A: Secretion of the PTHrP protein from EGR4-overexpressing HEK293T cells (n = 3, *P<0.05). B: Measurement scheme for the paracrine effects of conditioned media from EGR4-overexpressing cells. C: Real-time PCR analysis of the paracrine effects on the expression of the *RANKL, IL-6* and *IL-8* genes when medium from EGR4-overexpressing HEK293T cells was cultured with mouse MC3T3-E1 osteoblast cells (n = 2, *, *P*<0.05, **, *P*<0.01).

It has been reported that PTHrP protein secreted from cancer cells regulates the expression of the *RANKL*, *IL-6* and *IL-8* genes, which have been implicated as factors that enhance osteoclast formation and bone destruction in malignant diseases [28]–[30] in osteoblast cells. According to these data and our findings as shown in Figure 1, we hypothesized that PTHrP protein is secreted from EGR4-overexpressing cells. Our results showed that the PTHrP protein concentration was significantly increased in media conditioned from EGR4-overexpressing HEK293T cells (14.43±1.04 pmol/L) compared with conditioned media from mock-transfected cells (11.83±0.15 pmol/L, *P*<0.05; Figure 2A).

Next, we evaluated the paracrine effects of conditioned medium from EGR4-overexpressing HEK293T cells on osteoblast cells. As shown in Figure 2B, we transferred conditioned medium from HEK293T cells transfected with the FLAG-EGR4 construct to MC3T3-E1 murine osteoblast cells and then performed real-time PCR to examine the effects of the conditioned medium on the expression level of the *RANKL*, *IL-6* and *IL-8* genes. All three genes were significantly upregulated in osteoblast cells treated with conditioned medium from HEK293T cells ectopically expressing FLAG-EGR4 compared with mock-transfected cells (Figure 2C). Collectively, these findings suggest that the increase in PTHrP secretion from EGR4-overexpressing cells may enhance the expression of the *RANKL*, *IL-6* and *IL-8* genes in osteoblast cells.

### Effect of *EGR4* on cell growth

**Figure 3:**
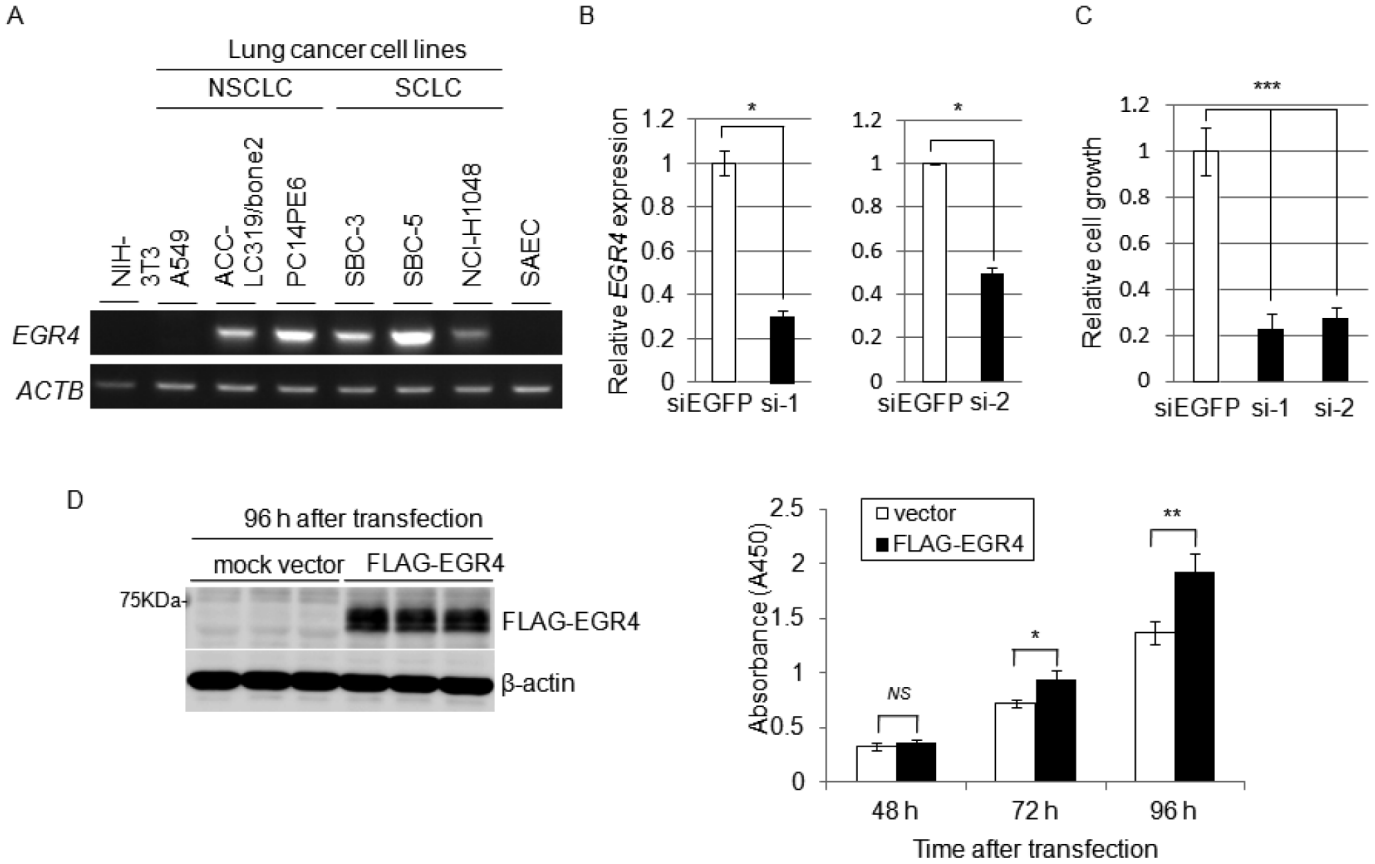
Effects of *EGR4* gene on cell growth. A: Expression of *EGR4* in SCLC and NSCLC cell lines was determined by semi-quantitative RT-PCR. B: Effects of *EGR4* knockdown on cell proliferation in SBC-5 cells. Real-time PCR of *EGR4* in siEGFP- or siEGR4 (siEGR4-1, siEGR4-2)-treated cells at 5 days after siRNA treatment (n = 2, *P<0.05). *ACTB* was used as a quantitative control for real-time RT-PCR. C: Cell proliferation was determined by MTT assay at 5 days after siRNA treatment (n = 3, ***P<0.005). (si-1; siEGR4-1, si-2; siEGR4-2). D: Growth–promoting effect of exogenous EGR4 on NIH3T3 cells (*P<0.05, **P<0.01, *NS*, no significance). Western blot analysis was performed at 96 h after transfection (left panel). MTT assay was performed at 48, 72 and 96 h after transfection with FLAG-EGR4 (black) or mock vector (white) (right panel). These experiments were performed in triplicate.

We first examined *EGR4* expression in SCLC cells by semi-quantitative RT-PCR and found that *EGR4* was highly expressed in SBC-3, SBC-5 and NCI-H1048 cells but not in the small airway epithelial cell line SAEC (Figure 3A). Next, to assess whether EGR4 is essential for the growth of SBC-5 cells, we used an RNA interference approach with two different siRNA oligonucleotides. Real-time PCR analysis showed that *EGR4*-specific siRNAs (siEGR4-1 and siEGR4-2) significantly suppressed the expression of EGR4 compared with siEGFP as a control (Figure 3B). MTT assay showed that the introduction of siEGR4s (siEGR4-1 and siEGR4-2) significantly suppressed the growth of SBC-5 cells (Figure 3C), which is in accordance with the EGR4 knockdown results. We also confirmed significant growth inhibitory effects of *EGR4* knockdown in other SCLC cell lines SBC-3 and NCI-H1048 overexpressing *EGR4* (Figure S1). To further confirm the growth promoting effect of *EGR4*, FLAG-EGR4 construct or mock vector was transiently transfected into NIH3T3 cells, and MTT assay was performed as described above. As shown in Figure 3D, FLAG-EGR4-transfected cells grew significantly faster than those transfected with mock vector. These findings suggest that overexpression of *EGR4* might be involved in the growth of SCLC cells.

### Identification of *EGR4* target genes

**Figure 4:**
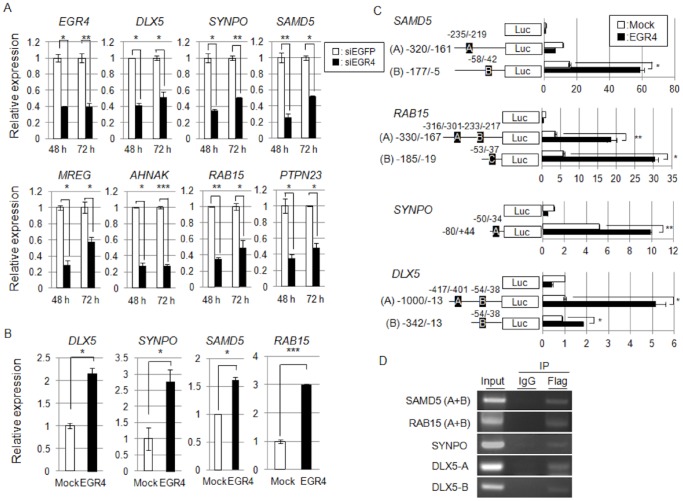
Identification of EGR4-downstream genes involved in the proliferation of SCLC cells. A: Real-time PCR of *EGR4* and seven downstream genes (*DLX5, SYNPO, SAMD5*, *MREG, AHNAK, RAB15*, and *PTPN23*) in siEGFP- or siEGR4-treated SBC-5 cells (n = 2, *, *P*<0.05, **, *P*<0.01, ***, *P*<0.005). B: Real-time PCR of the *DLX5*, *SYNPO*, *SAMD5*, and *RAB15* genes in mock- or EGR4-overexpressing HEK293T cells (n = 2, *, *P*<0.05, ***, *P*<0.005). This experiment was performed using total RNA from cells expressing exogenous FLAG-tagged EGR4 (FLAG-EGR4) or those transfected with the mock vector used in Figure 1B. C: Luciferase assay of the *SAMD5*, *RAB15*, *SYNPO* and *DLX5* genes. (n = 2, *, *P*<0.05, **, *P*<0.01). D: ChIP assays were used to determine the direct binding of EGR4 to the promoters of the *SAMD5*, *RAB15*, *SYNPO* and *DLX5* genes.

To obtain further insight into the biological role of *EGR4* on cell growth, we attempted to identify downstream genes specifically regulated by EGR4 in SCLC cells. siEGR4 or siEGFP (control siRNA) was transfected into SBC-5 cells in which *EGR4* was highly expressed (Figure 3A), and alterations in gene expression at two time points were monitored by DNA microarray analysis. To identify the genes putatively regulated by EGR4, we selected genes with the following two criteria: (i) expression level was decreased by more than two-fold at 48 and 72 h in cells transfected with siEGR4 compared with cells transfected with the control siEGFP, and (ii) a putative EGR binding motif was predicted to exist within 500 bp of the transcription start site by the MatInspector program (described above). We identified 13 genes that were downregulated upon knockdown of EGR4 expression (Table S5). Real-time PCR analysis confirmed that seven transcripts were significantly downregulated at both time points in EGR4-knockdown cells (Figure 4A). Subsequently, we also evaluated the upregulation of these genes upon exogenous EGR4 expression in HEK293T cells and ultimately selected four EGR4 candidate target genes, including distal-less homeobox 5 (*DLX5*), synaptopodin (*SYNPO*), sterile alpha motif domain containing 5 (*SAMD5*), and RAB15, a member of the RAS oncogene family (*RAB15*), which were significantly upregulated by *EGR4* overexpression (Figure 4B). We confirmed significant downregulation of *DLX5*, *SYNPO* and *SAMD5* genes by *EGR4* knockdown in SBC-3 cells (Figure S2).

**Figure 5:**
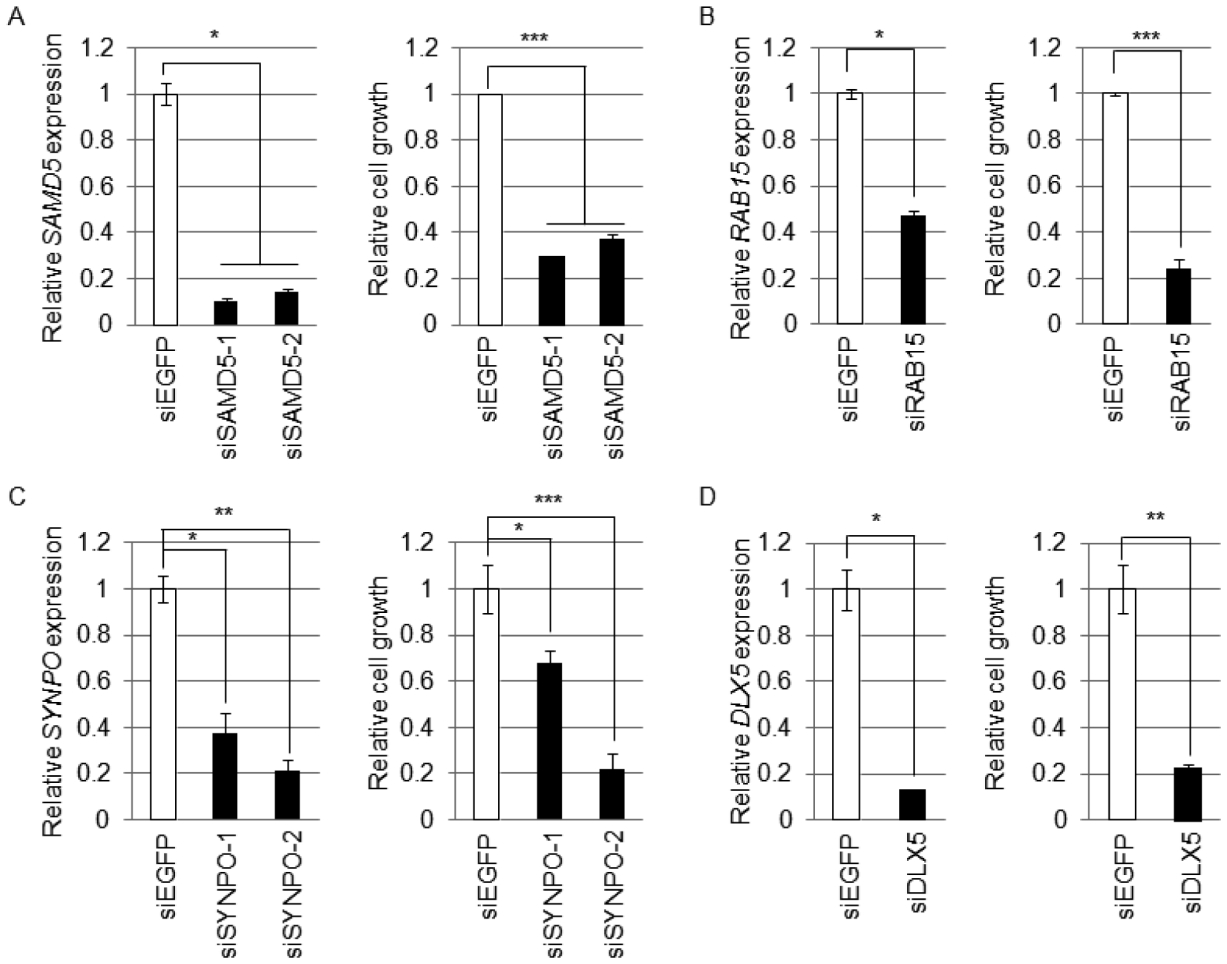
*EGR4* downstream target genes regulate the cell proliferation of SBC-5 cells. Effects of the *EGR4* downstream genes *SAMD5* (A), *RAB15* (B), *SYNPO* (C) and *DLX5* (D) on cell proliferation were determined by siRNA knockdown in SBC-5 cells. The left panel shows the real-time PCR results for target genes in siRNA-treated cells (n = 2). The right panel shows results from cell proliferation analyses as measured by MTT assay (*SAMD5* and *RAB15*: n = 2, *DLX5* and *SYNPO*: n = 3, *, *P*<0.05, **, *P*<0.01, ***, *P*<0.005).

To obtain direct evidence for the transactivation of four EGR4 candidate target genes, we measured the transcriptional activity of EGR4 by a luciferase reporter assay. FLAG-EGR4-tranfected cells had significantly higher luciferase activity than mock-transfected cells (Figure 4C). Next, we investigated the recruitment of EGR4 to each EGR4-binding site by ChIP assay. EGR4 was shown to bind to the predicted EGR-binding motif within the promoter regions of all target genes (Figure 4D). These results suggest that EGR4 directly transactivates *SAMD5*, *RAB15*, *SYNPO* and *DLX5*. Subsequently, we investigated the biological role of the four EGR4 target genes in the proliferation of SCLC cells. Introduction of the siRNAs into SBC-5, SBC-3 and NCI-H1048 cells resulted in a significant reduction in the expression of the target genes accompanied by significant suppression of cell proliferation (Figure 5A–D, Figure S3), suggesting that these genes are also likely to play a crucial role in the proliferation of SCLC cells via EGR4 transcriptional activation.

## Discussion

In this study, our aim was to identify and characterize molecules or pathways potentially involved in cancer metastasis, particularly bone metastasis. Through a genome-wide transcriptomic analysis of the organ-preferential metastasis of human SCLC cells in mice, we found that *EGR4*, a member of a family of four related zinc-finger Cys_2_-His_2_ type proteins (*EGR1* to *EGR4*), is significantly upregulated in bone metastatic tumors compared with other organs i.e., the lung, kidney and liver [7]. EGR4 was initially identified as a zinc-finger transcription factor immediately induced by mitogenic stimulation in T lymphocytes and fibroblasts [31]. Gene targeting studies in mice have shown that EGR4 regulates several critical genes involved in the early stages of meiosis and plays an indispensable role in male murine fertility [11], [12]. Furthermore, it has been reported that EGR4 binds to nuclear factor activated T cells (NFAT) or nuclear factor kappa B (NFκB) to enhance the transcription of downstream genes encoding inflammatory cytokines, such as IL-2, TNF-α and ICAM-1 [32], [33]. A previous report described that the expression level of *PTHrP*, a potent activator of osteoclastic bone resorption, in bone metastases tends to be higher than that in metastases to the kidneys, livers, and lungs using a genome-wide transcriptomics of human SCLC cells in mice [7]. Accordingly, in this study, we focused on *PTHrP*, a potent activator of osteoclastic bone resorption, as an EGR4-downstream gene to clarify the pathophysiological role of EGR4 as a transcription factor in SCLC bone metastases.

PTHrP is known to be a key mediator of humoral hypercalcemia malignancies and osteolytic lung cancer metastases [22], [23], [34]. Approximately 80% of patients with solid tumors and hypercalcemia have increased PTHrP concentrations in their plasma [35]. It has been reported that PTHrP protein secreted from cancer cells regulates the expression of the *RANKL*, *IL-6* and *IL-8* genes, which have been implicated as factors that enhance osteoclast formation and bone destruction in malignant diseases [28]–[30] in osteoblast cells. We found that EGR4 directly transactivates specific variants (*V3* and *V4*) of the *PTHrP* gene, thereby possibly promoting the secretion of the PTHrP protein in EGR4-overexpressing cells, resulting in subsequent transactivation of the *RANKL*, *IL-6* and *IL-8* genes via paracrine action of PTHrP. RANKL is known to bind the RANK receptor on osteoclast precursors and induce osteoclast formation. IL-6 and IL-8 have also been reported to be important for osteoclastogenesis and osteoclast activation, respectively [30]. Therefore, these findings suggest that induction of PTHrP by EGR4 overexpression may be responsible for the bone metastasis of SCLC lung cancer cells. However, we found that *PTHrP* gene expression was not reduced by EGR4 knockdown in SBC5 cells (data not shown). A possible reason for this result is that several factors are involved in the regulation of *PTHrP* expression in addition to the EGR4 transcription factor. For example, EGR4 is reported to functionally cooperate with NF-κB and NFAT and induce the expression of cytokine genes [32], [33]. Indian hedgehog and TGF-β have also been reported to stimulate perichondrial and breast cancer production, respectively [36], [37]. Moreover, miR-33a has been reported to repress the PTHrP-mediated expression of *PTHrP* in NSCLC [38], and knockdown of zinc-finger E-box binding homeobox 1 (ZEB1), a transcriptional repressor, reduces PTHrP secretion in SCLC [39]. Therefore, it is necessary to further explore the mechanism of *PTHrP* transactivation via endogenous EGR4 expression in SBC-5 cells in greater detail.

Notably, we showed that depletion of *EGR4* by siRNA led to a significant reduction in cell proliferation in SBC-5, SBC-3 and NCI-H1048 cells, and that EGR4 transactivated a set of genes possibly related to lung cancer cell growth including four EGR4-downstream genes, *DLX5*, *RAB15, SAMD5* and *SYNPO*. Knockdown of the expression of these genes by siRNA led to a significant reduction in cell growth in SCLC cells, suggesting that these genes are involved in the growth of SCLC lung cancer cells. It has been reported that *DLX-5* overexpression in lung cancer cells is associated with tumor size and predictive of poor prognosis and NSCLC cell proliferation [20]. RAB15 was originally identified as a brain-tissue specific RAB protein within the RAB family of small G proteins that regulates the endocytic recycling pathway [40] and is associated with the retinoic acid-induced differentiation of neuroblastoma cells [41]. *SAMD5* has been reported to be one of 24 discriminating genes with an expression level that significantly differs between responders and nonresponders to chemoradiotherapy in rectal cancer [42]. SYNPO has been reported to be an actin-binding protein that functions in actin dynamics, cell migration, and tumor suppression [43] and is exclusively expressed in highly dynamic cell compartments such as kidney podocyte foot processes [44]. Although the precise function of these genes in lung carcinogenesis remains largely unknown, our findings suggest that EGR4 may be a pivotal regulator that selectively activates the transcription of several target genes in lung cancer cells.

In addition, we demonstrated that *EGR4* was highly expressed in NSCLC and SCLC cell lines (Figure 3A). In addition, analysis of publicly available RNAseq data sets from The Cancer Genome Atlas (TCGA) revealed that *EGR4* was up-regulated (more than 2-fold) in 17 of 39 lung adenocarcinoma cases (Figure S4A), and in 19 of 46 squamous cell carcinoma (SCC) cases (Figure S4B) compared with their corresponding normal lung. Furthermore, we found that knockdown of *EGR4* by siRNA suppressed the proliferation of PC14PE6 NSCLC cells (Figure S5), but did not find the inhibitory effects of EGR4 knockdown on its downstream genes, *SAMD5*, *RAB15*, *SYNPO* and *DLX5* expression in PC14PE6 cells (data not shown). These findings suggest the possibility that EGR4 may play different roles in NSCLC cell growth. Therefore, it is necessary to further explore the mechanism of *EGR4* transactivation in NSCLC cells.

In summary, we demonstrated that EGR4 directly transactivates specific variants (*V3* and *V4)* of the *PTHrP* gene, thereby possibly enhancing secretion of the PTHrP protein in EGR4-overexpressing cells, resulting in subsequent transactivation of the *RANKL*, *IL-6* and *IL-8* genes via paracrine action of the PTHrP protein, a mediator of osteolytic bone metastasis (Figure 2). Moreover, EGR4 also transactivates *SAMD5*, *RAB15*, *SYNPO* and *DLX5*, which are involved in the proliferation of SCLC cells. Collectively, our findings suggest that EGR4 is likely to play an important role for the promotion of SCLC growth through the up-regulation of its downstream genes, and it could be a novel therapeutic target for the development of anticancer drugs.

## Supporting Information

Figure S1 **Effects of** ***EGR4*** **gene on SBC-3 and NCI-H1048 cell growth.** Effects of *EGR4* knockdown on cell proliferation in SBC-3 and NCI-H1048 cells. Real-time PCR of *EGR4* in siEGFP- or siEGR4 (siEGR4-1, siEGR4-2)-treated SBC-3 cells (A) and NCI-H1048-cells (C) at 5 days after siRNA treatment (n = 2, *P<0.05, **, *P*<0.01). *ACTB* was used as a quantitative control for real-time RT-PCR. Cell proliferation of SBC-3 (B) and NCI-H1048 (D) was determined by MTT assay at 5 days after siRNA treatment (n = 3, *P<0.05, **, *P*<0.01, ***P<0.005). (si-1; siEGR4-1, si-2; siEGR4-2).
(TIFF)

Figure S2 **Expression of EGR4-downstream genes in siEGR4-treated SBC-3 cells.** Real-time PCR of *EGR4* and 3 downstream genes (*DLX5, SYNPO* and *SAMD5*) in SBC-3 cells treated with siEGFP or siEGR4 for 48 h (n = 2, *, *P*<0.05, **, *P*<0.01, ***, *P*<0.005).
(TIFF)

Figure S3 ***EGR4*** **-downstream target genes regulate the cell proliferation of SBC-3 and NCI-H1048 cells.** Effects of the *EGR4* downstream genes on cell proliferation were determined in SBC-3 (A–C) and NCI-H1048 cells (D, E). The left panel shows the real-time PCR results for *EGR4*-downstream genes in siRNA-treated cells (n = 2). The right panel shows results from cell proliferation analyses as measured by MTT assay (n = 3, *, *P*<0.05, **, *P*<0.01, ***, *P*<0.005).
(TIF)

Figure S4 **Overexpression of** ***EGR4*** **in lung cancers.** The cases overexpressing *EGR4* (>2 fold compared with the adjacent normal lung) are indicated as black bars in adenocarcinoma (A) and squamous cell carcinoma (SCC) (B).
(TIFF)

Figure S5 **Effect of** ***EGR4*** **on cell proliferation in PC14PE6 cells.** Knockdown of *EGR4* at the mRNA level was analyzed by real-time PCR (n = 2, **P<0.01, ***P<0.005). Cell proliferation was determined by an MTT assay at 4 days after siRNA treatment (n = 3, ***, *P*<0.005).
(TIFF)

Table S1 **Primer sequences for plasmid construction.**
(DOCX)

Table S2 **Primer sequences for real time PCR or RT-PCR.**
(DOCX)

Table S3 **Primer sequences for ChIP assay.**
(DOCX)

Table S4 **siRNA sequences.**
(DOCX)

Table S5 **Putative downstream EGR4 target genes identified by microarray analysis.**
(DOCX)
