# Supplementary figures and images for: Early Growth Response 4 Is Involved in Cell Proliferation of Small Cell Lung Cancer through Transcriptional Activation of Its Downstream Genes
Source: PLoS One. 2014 Nov 20;9(11):e113606. doi: 10.1371/journal.pone.0113606 (PMC4239076; doi:10.1371/journal.pone.0113606)

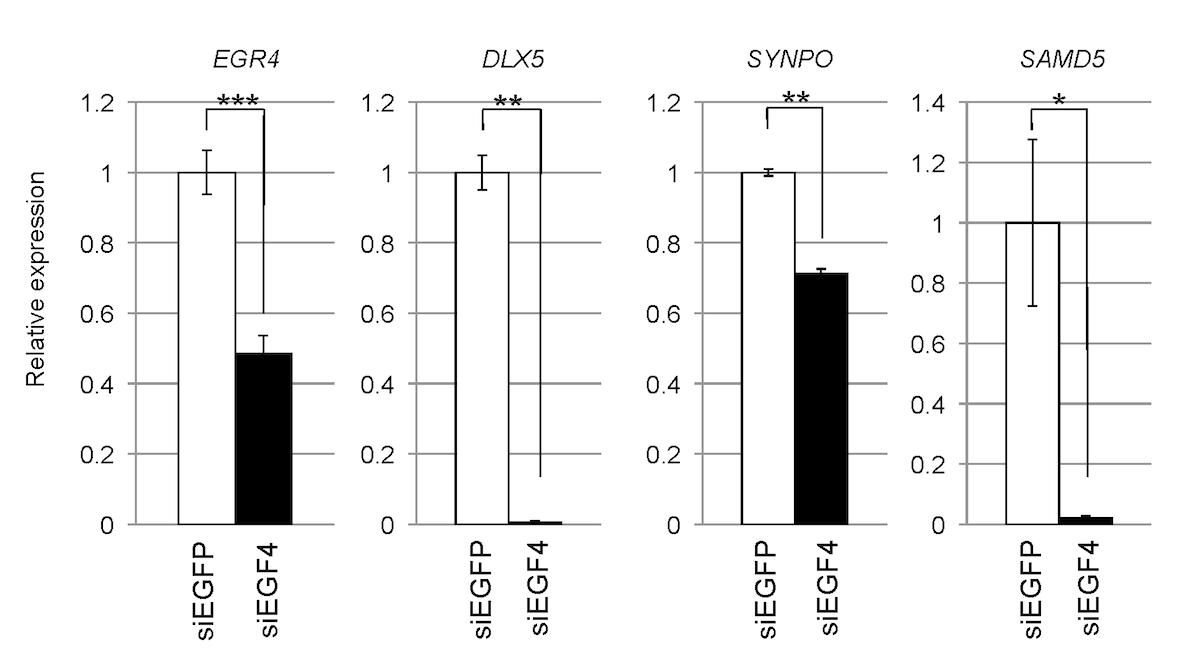

Supplement: Figure S2 — Expression of EGR4-downstream genes in siEGR4-treated SBC-3 cells. Real-time PCR of EGR4 and 3 downstream genes (DLX5, SYNPO and SAMD5) in SBC-3 cells treated with siEGFP or siEGR4 for 48 h (n = 2, *, P<0.05, **, P<0.01, ***, P<0.005). (TIFF) [file pone.0113606.s002.tiff]

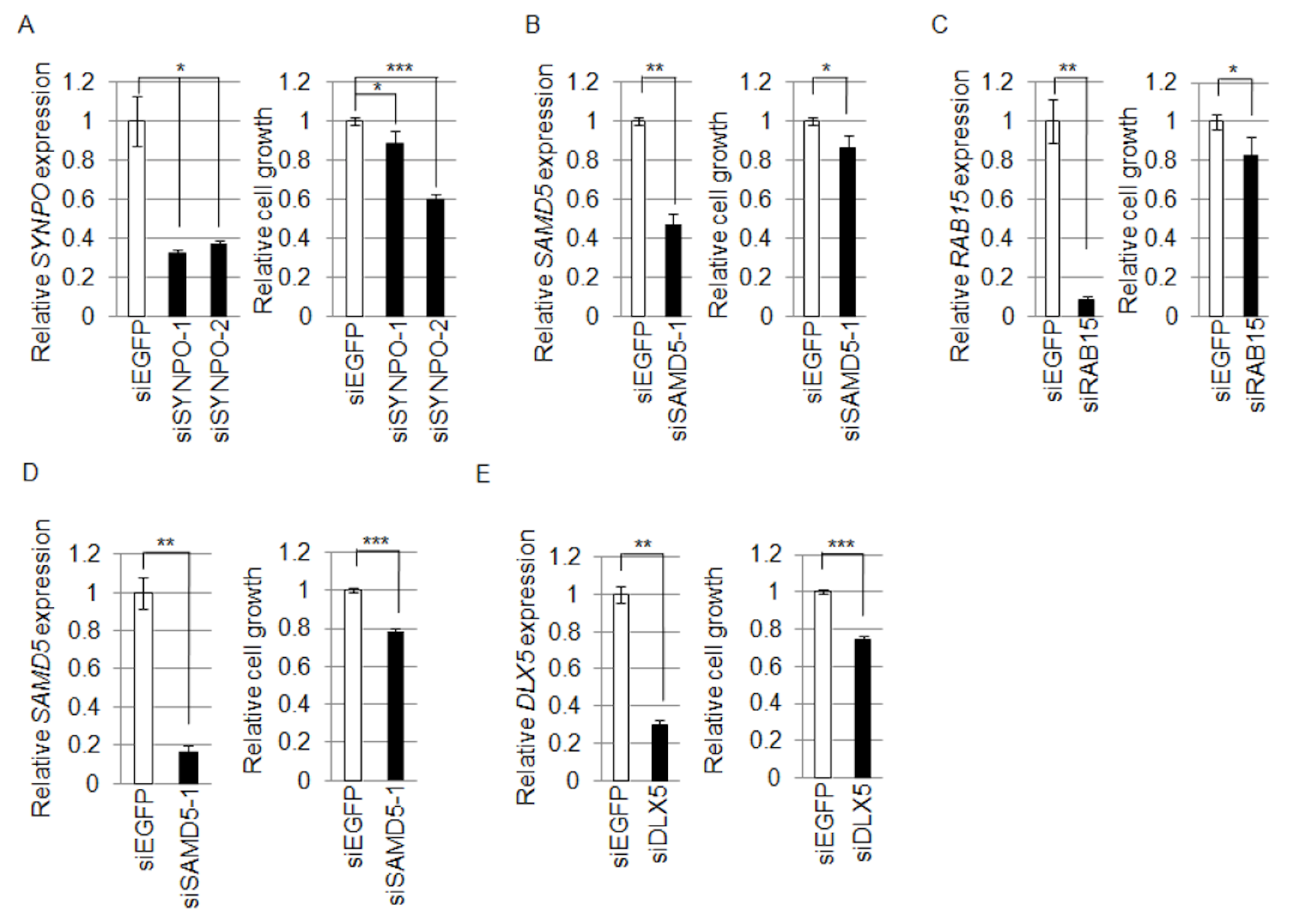

Supplement: Figure S3 — EGR4 -downstream target genes regulate the cell proliferation of SBC-3 and NCI-H1048 cells. Effects of the EGR4 downstream genes on cell proliferation were determined in SBC-3 (A–C) and NCI-H1048 cells (D, E). The left panel shows the real-time PCR results for EGR4-downstream genes in siRNA-treated cells (n = 2). The right panel shows results from cell proliferation analyses as measured by MTT assay (n = 3, *, P<0.05, **, P<0.01, ***, P<0.005). (TIF) [file pone.0113606.s003.tif]

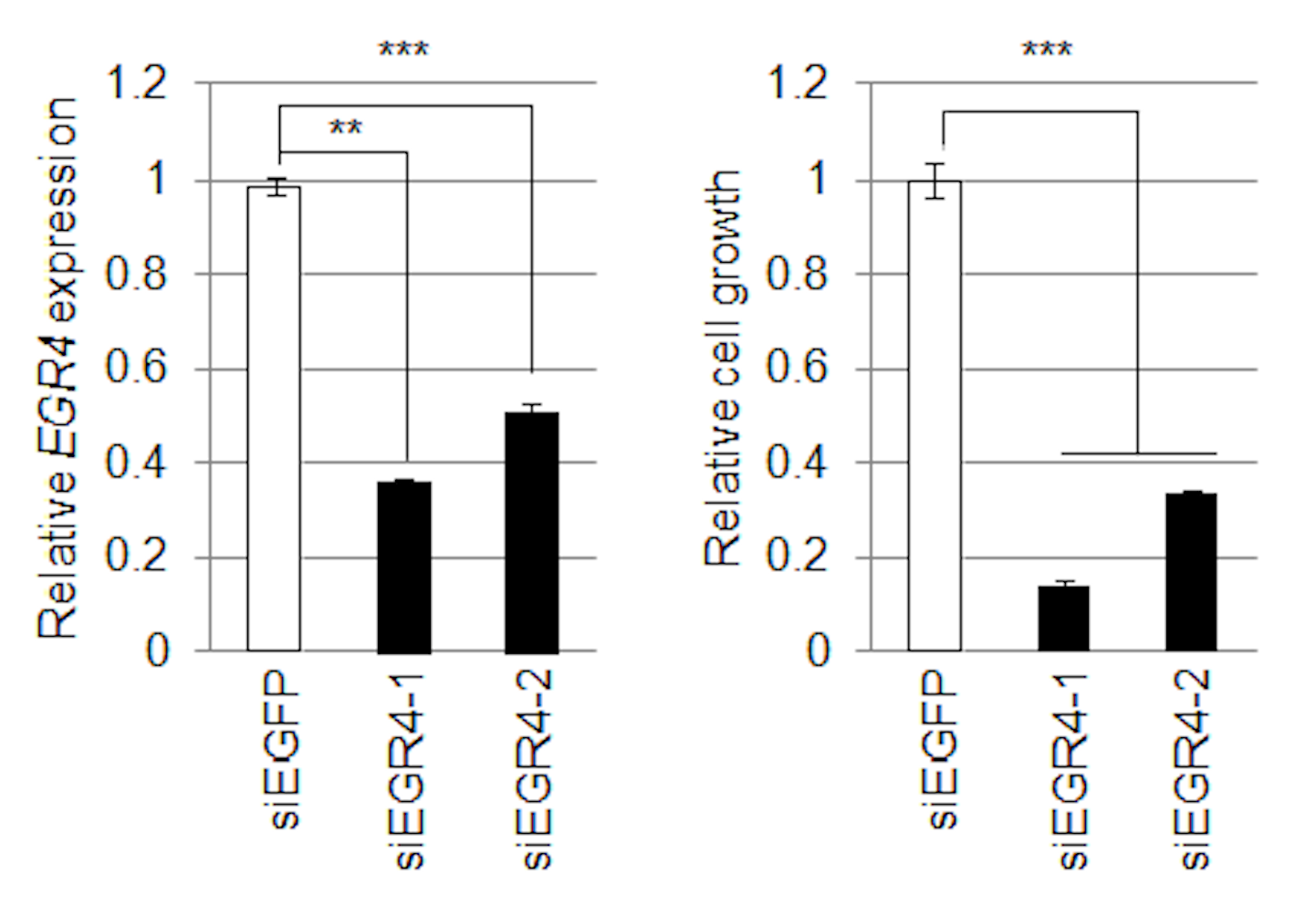

Supplement: Figure S5 — Effect of EGR4 on cell proliferation in PC14PE6 cells. Knockdown of EGR4 at the mRNA level was analyzed by real-time PCR (n = 2, **P<0.01, ***P<0.005). Cell proliferation was determined by an MTT assay at 4 days after siRNA treatment (n = 3, ***, P<0.005). (TIFF) [file pone.0113606.s005.tiff]
