# Supplementary material for: Early Growth Response 4 Is Involved in Cell Proliferation of Small Cell Lung Cancer through Transcriptional Activation of Its Downstream Genes
Source: PLoS One. 2014 Nov 20;9(11):e113606. doi: 10.1371/journal.pone.0113606 (PMC4239076; doi:10.1371/journal.pone.0113606)
Supplement: Table S1 — Primer sequences for plasmid construction. (DOCX) [file pone.0113606.s006.docx]

Table S1. Primer sequences for plasmid construction

| gene name | type | primer sequence |
| --- | --- | --- |
| EGR4 | forward | CGGAATTCATGCTCCACCTTAGCGAGT |
| EGR4 | reverse | CCGCTCGAGTCAGAGAGAAGCGAAGGAGA |
| PTHrP(-621/-318) | forward | GGGGTACCTGGAGGGAGCAAGCGGATG |
| PTHrP(-621/-318) | reverse | CCCAAGCTTGTGAGCTAGTCGCAAAGAG |
| SAMD5(-320/-161) | forward | GGGGTACCACAGTGACTGACACTCGCG |
| SAMD5(-320/-161) | reverse | CCCAAGCTTCCGCGAGTTTTCCCAGC |
| SAMD5(-177/-5) | forward | GGGGTACCGCTGGGAAAACTCGCGG |
| SAMD5(-177/-5) | reverse | CCCAAGCTTCTGCTGGGCTGAGCAAGG |
| RAB15(-330/-167) | forward | GGGGTACCGCAGGAAGGGGTTGGGAGG |
| RAB15(-330/-167) | reverse | CCCAAGCTTGCGGTGGGAGGAGAAACCG |
| RAB15(-185/-19) | forward | GGGGTACCCGGTTTCTCCTCCCACCGC |
| RAB15(-185/-19) | reverse | CCCAAGCTTCGGCGAGGAGGACGCC |
| SYNPO(-80/+44) | forward | CCGCTCGAGAAGATGAATCTGGCAGCGTG |
| SYNPO(-80/+44) | reverse | GAAGATCTCCACAGATGACTCAGCCCA |
| DLX5(-1000/-13) | forward | CCGCTCGAGTTGCAAAGGCTTTGATCCTT |
| DLX5(-342/-13) | forward | CCGCTCGAGGGCTTCTGATTGGAACACAT |
| DLX5(-1000, -342/-13) | reverse | GAAGATCTGCACAGCCTTGGTTAAATCC |

The restriction enzyme sites have been underlined.
