# Supplementary material for: Early Growth Response 4 Is Involved in Cell Proliferation of Small Cell Lung Cancer through Transcriptional Activation of Its Downstream Genes
Source: PLoS One. 2014 Nov 20;9(11):e113606. doi: 10.1371/journal.pone.0113606 (PMC4239076; doi:10.1371/journal.pone.0113606)
Supplement: Table S2 — Primer sequences for real time PCR or RT-PCR. (DOCX) [file pone.0113606.s007.docx]

Table S2. Primer sequences for real time PCR or RT-PCR

| gene name | type | primer sequence | experiment |
| --- | --- | --- | --- |
| EGR4 | forward | AGCAAGAGATGGGTTTATG | semi-quantitative and real time PCR  (figure 3, 4, S1, S2, and S5) |
| EGR4 | reverse | AGGAGTTGGAAGAAGAGC | semi-quantitative and real time PCR  (figure 3, 4, S1, S2, and S5) |
| EGR4 | forward | TTGAGCTGGGCTTTGAACA | real time PCR (for siEGR4-1 in figure 3, S1, and S5) |
| EGR4 | reverse | AGATGCCCGACATGAGGTT | real time PCR (for siEGR4-1 in figure 3, S1, and S5) |
| PTHrP-V1/V2 | reverse | CGACACACGCACTTGAAACT | real time PCR |
| PTHrP-V1/V2 | forward | CCACTGCTGAACCAGTCTCC | real time PCR |
| PTHrP-V3/V4 | reverse | AGAGGAAGCGCCTCTGATTT | real time PCR |
| PTHrP-V3/V4 | forward | CGCTCGGGACTTATTTAGCA | real time PCR |
| SAMD5 | forward | CATGGGCCTTGGAAAATCTA | real time PCR (for expression analysis in figure 4 and S2) |
| SAMD5 | reverse | GGCATGCATTACATGGTTCA | real time PCR (for expression analysis in figure 4 and S2) |
| SAMD5 | forward | TGTTCTGAGCTGCAGTGCTT | real time PCR (for siRNA analysis in figure 5 and S3) |
| SAMD5 | reverse | GCATCCAAACACAAGAACGA | real time PCR (for siRNA analysis in figure 5 and S3) |
| RAB15 | forward | GCCATGACTGGGAAAAGGTA | real time PCR (for expression analysis in figure 4) |
| RAB15 | reverse | AAGACAGCAGGTGCAGAGGT | real time PCR (for expression analysis in figure 4) |
| RAB15 | forward | GGCTGATGAGGAGCAGAAAC | real time PCR (for siRNA analysis in figure 5 and S3) |
| RAB15 | reverse | GCCTTCCAGCTCCTTCCTAT | real time PCR (for siRNA analysis in figure 5 and S3) |
| SYNPO | forward | GCCTGGCTGTTCTCTCATT | real time PCR |
| SYNPO | reverse | GCCAGAGCTGCATTCTACA | real time PCR |
| DLX5 | forward | TGGAGAACTCTGCATCCTGGTA | real time PCR |
| DLX5 | reverse | CGGCAGGTGGGAATTGATT | real time PCR |
| hACTB | forward | ATTGCCGACAGGATGCAG | real time PCR |
| hACTB | reverse | CTCAGGAGGAGCAATGATCTT | real time PCR |
| mhACTB | forward | CCTCCCTGGAGAAGAGCTA | RT-PCR |
| mhACTB | reverse | TTGCTGATCCACATCTGCTG | RT-PCR |
| mRANKL | forward | CGTAGCTAAGGGGGCAGA | real time PCR |
| mRANKL | reverse | GCACCTGGTGACCAACAT | real time PCR |
| mIL-6 | forward | ACCCCAATTTCCAATGCT | real time PCR |
| mIL-6 | reverse | CCACAGTGAGGAATGTCCA | real time PCR |
| mIL-8 | forward | CCATTGCCCAGATGTTGT | real time PCR |
| mIL-8 | reverse | CACCCTCTCCCCAGAAAC | real time PCR |

h;human, m; mouse
