# Supplementary material for: Early Growth Response 4 Is Involved in Cell Proliferation of Small Cell Lung Cancer through Transcriptional Activation of Its Downstream Genes
Source: PLoS One. 2014 Nov 20;9(11):e113606. doi: 10.1371/journal.pone.0113606 (PMC4239076; doi:10.1371/journal.pone.0113606)
Supplement: Table S3 — Primer sequences for ChIP assay. (DOCX) [file pone.0113606.s008.docx]

Table S3. Primer sequences for ChIP assay

| gene name | type | primer sequence |
| --- | --- | --- |
| PTHrP(-621/-318) | forward | TGGAGGGAGCAAGCGGATG |
| PTHrP(-621/-318) | reverse | GTGAGCTAGTCGCAAAGAG |
| SAMD5(-317/+70) | forward | GTGACTGACACTCGCGAAGA |
| SAMD5(-317/+70) | reverse | AAGCCTGAGGAAGGGAGAAG |
| RAB15(-391/+96) | forward | CTCACCTCAACTCCCCCATT |
| RAB15(-391/+96) | reverse | GTACTGCTTCGCCATGACTG |
| SYNPO(-180/+32) | forward | GAATGCCAGGATGAGGACT |
| SYNPO(-180/+32) | reverse | CAGCCCAGCTCTTTATCAGG |
| DLX5(-439/-293)-A | forward | TTCTACACTCGCCTTTGGTG |
| DLX5(-439/-293)-A | reverse | CAGCACAAGGCTCTGTGATG |
| DLX5(-238/-13)-B | forward | CCCACTCCACAACAAGCAA |
| DLX5(-238/-13)-B | reverse | GCACAGCCTTGGTTAAATCC |
