# Supplementary material for: Early Growth Response 4 Is Involved in Cell Proliferation of Small Cell Lung Cancer through Transcriptional Activation of Its Downstream Genes
Source: PLoS One. 2014 Nov 20;9(11):e113606. doi: 10.1371/journal.pone.0113606 (PMC4239076; doi:10.1371/journal.pone.0113606)
Supplement: Table S4 — siRNA sequences. (DOCX) [file pone.0113606.s009.docx]

Table S4. siRNA sequences

| gene | siRNA sequence |
| --- | --- |
| siEGR4-1 | GCUACAGCGGUAGCUUCUU |
| siEGR4-2 | GGACCAAGAUUGAGGACUU |
| siSAMD5-1 | GCUAAGGCAUUUGAGUCAU |
| siSAMD5-2 | GGACUAAUACAAGUGUCUU |
| siRAB15 | CCAUAGAGGUAGACGGCAU |
| siSYNPO-1 | GGCGCGAAACAUCAUCAAU |
| siSYNPO-2 | CCAGAGAAGCUACGCUCAU |
| siDLX5 | GUGCAGCCAGCUCAAUCAA |
| siEGFP | GCAGCACGACUUCUUCAAG |
