# Supplementary material for: Early Growth Response 4 Is Involved in Cell Proliferation of Small Cell Lung Cancer through Transcriptional Activation of Its Downstream Genes
Source: PLoS One. 2014 Nov 20;9(11):e113606. doi: 10.1371/journal.pone.0113606 (PMC4239076; doi:10.1371/journal.pone.0113606)
Supplement: Table S5 — Putative downstream EGR4 target genes identified by microarray analysis. (DOCX) [file pone.0113606.s010.docx]

Table S5. Putative downstream EGR4 target genes identified by microarray analysis

| Probe ID | Accession no. | Symbol | Gene Name | FC  48h | *P*-value | FC  72h | *P*-value |
| --- | --- | --- | --- | --- | --- | --- | --- |
| A_23_P108948 | NM_018000 | *MREG* | melanoregulin | -3.12 | 5.62E-03 | -2.51 | 1.40E-03 |
| A_23_P21363 | NM_024060 | *AHNAK* | AHNAK nucleoprotein | -2.70 | 4.64E-03 | -3.04 | 1.33E-03 |
| A_24_P602871 | NM_001030060 | *SAMD5* | sterile alpha motif domain containing 5 | -2.50 | 1.37E-02 | -2.15 | 6.47E-03 |
| A_24_P193295 | NM_198686 | *RAB15* | RAB15, member RAS onocogene family | -2.44 | 1.93E-03 | -2.01 | 1.04E-02 |
| A_24_P328675 | NM_015466 | *PTPN23* | protein tyrosine phosphatase, non-receptor type 23 | -2.36 | 2.02E-03 | -2.17 | 1.58E-03 |
| A_23_P86195 | NM_152369 | *SLC44A3* | solute carrier family 44, member 3 | -2.34 | 3.66E-03 | -2.01 | 9.50E-03 |
| A_23_P77103 | NM_003104 | *SORD* | sorbitol dehydrogenase | -2.34 | 3.68E-03 | -2.22 | 3.59E-03 |
| A_23_P344531 | NM_007286 | *SYNPO* | synaptopodin | -2.32 | 2.56E-03 | -2.01 | 2.01E-03 |
| A_32_P89691 | NM_003104 | *SORD* | sorbitol dehydrogenase | -2.30 | 4.97E-03 | -2.19 | 4.09E-04 |
| A_32_P127153 | NM_003104 | *SORD* | sorbitol dehydrogenase | -2.27 | 1.02E-03 | -2.12 | 4.45E-03 |
| A_24_P179316 | NM_015466 | *PTPN23* | protein tyrosine phosphatase, non-receptor type 23 | -2.27 | 8.56E-03 | -2.01 | 1.20E-02 |
| A_23_P386561 | NM_001002926 | *TWISTNB* | TWIST neighbor | -2.26 | 4.19E-03 | -2.32 | 8.88E-04 |
| A_23_P213518 | NM_001042440 | *CAST* | calpastatin | -2.25 | 3.41E-03 | -2.11 | 3.49E-03 |
| A_23_P212329 | NM_015466 | *PTPN23* | protein tyrosine phosphatase, non-receptor type 23 | -2.21 | 1.79E-03 | -2.24 | 2.76E-04 |
| A_32_P187009 | NM_001174072 | *SERINC5* | serine incorporator 5 | -2.18 | 2.41E-03 | -2.13 | 3.02E-03 |
| A_23_P82474 | NM_001002926 | *TWISTNB* | TWIST neighbor | -2.08 | 1.23E-02 | -2.08 | 1.96E-03 |
| A_23_P132595 | NM_014667 | *VGLL4* | vestigial like 4 (Drosophila) | -2.05 | 6.20E-03 | -2.02 | 1.27E-03 |
| A_23_P318262 | NM_005221 | *DLX5* | distal-less homeobox 5 | -2.05 | 2.03E-03 | -2.08 | 3.68E-03 |

*P*-value, Benjamini-Hochberg false discovery rate of random permutation test; fold change (FC), ratio of gene expression level between siEGFP and siEGR4; Gene symbol, accession number and gene name, exported from GeneSpring (from the NCBI databases).
